# Computational Screening of Approved Drugs for Inhibition of the Antibiotic Resistance Gene *mecA* in Methicillin-Resistant *Staphylococcus aureus* (MRSA) Strains

**DOI:** 10.3390/biotech12020025

**Published:** 2023-03-31

**Authors:** Benson Otarigho, Mofolusho O. Falade

**Affiliations:** 1 Department of Molecular Microbiology and Immunology, Oregon Health and Science University, Portland, OR 97239, USA; 2 Department of Biology, Transylvania University, Lexington, KY 40508, USA

**Keywords:** methicillin-resistant *Staphylococcus aureus*, afamelanotide, antibiotic resistance gene, MecA, vancomycin

## Abstract

Antibiotic resistance is a critical problem that results in a high morbidity and mortality rate. The process of discovering new chemotherapy and antibiotics is challenging, expensive, and time-consuming, with only a few getting approved for clinical use. Therefore, screening already-approved drugs to combat pathogens such as bacteria that cause serious infections in humans and animals is highly encouraged. In this work, we aim to identify approved antibiotics that can inhibit the *mecA* antibiotic resistance gene found in methicillin-resistant *Staphylococcus aureus* (MRSA) strains. The MecA protein sequence was utilized to perform a BLAST search against a drug database containing 4302 approved drugs. The results revealed that 50 medications, including known antibiotics for other bacterial strains, targeted the *mecA* antibiotic resistance gene. In addition, a structural similarity approach was employed to identify existing antibiotics for *S. aureus*, followed by molecular docking. The results of the docking experiment indicated that six drugs had a high binding affinity to the *mecA* antibiotic resistance gene. Furthermore, using the structural similarity strategy, it was discovered that afamelanotide, an approved drug with unclear antibiotic activity, had a strong binding affinity to the MRSA-MecA protein. These findings suggest that certain already-approved drugs have potential in chemotherapy against drug-resistant pathogenic bacteria, such as MRSA.

## 1. Introduction

Methicillin-resistant *Staphylococcus aureus* (MRSA) is a dangerous pathogenic strain of *Staphylococcus aureus* [1,2]. Compared to other *S. aureus* strains, such as methicillin-susceptible *S. aureus* (MSSA), vancomycin-intermediate *S. aureus* (VISA), and vancomycin-resistant *S. aureus* (VRSA), MRSA is the most challenging to treat due to its high virulence and resistance to certain antibiotics [3,4,5]. As the most prevalent antibiotic-resistant human pathogen, MRSA is a significant threat to healthcare systems [6,7,8,9,10,11,12,13,14]. In addition, MRSA poses a significant threat outside healthcare environments, leading to infections such as bacteremia or sepsis, pneumonia, endocarditis, and osteomyelitis. Approximately 2% of individuals carrying *S. aureus* in their nasal passages are infected with the MRSA strain out of the estimated 34% carrying the bacteria. Hundreds of thousands of cases and tens of thousands of deaths are reported yearly due to this bacterial infection [6]. Animals ranging from domesticated livestock to companion animals to captive or free-living wild terrestrial and/or aquatic species can also be colonized and infected by MRSA [15]. MRSA has been detected in various animal species such as horses, dogs, cats, cows, and pigs. The widespread use of antimicrobial agents in animal husbandry and other agricultural practices has greatly contributed to the broad distribution of MRSA among livestock [15,16]. In some cases, animals that have not received antibiotic treatment have been infected with MRSA, possibly due to transfer from humans to animals and vice versa. Studies have demonstrated that humans can also serve as a reservoir for transmitting *S. aureus* to vertebrate animals. Infections may exist in both humans and animals and can be transmitted in both directions. The role of animals in human MRSA infections varies widely depending on the animal species and geographical location [15,16,17].

Vancomycin is the gold-standard antibiotic used in the treatment of *S. aureus* infection [7,18], but there are other antibiotics such as daptomycin, linezolid, tigecycline, quinupristin/dalfopristin, tetracyclines, rifampin, gentamicin, and many others that have been found to have inhibitory effects on the bacterial infection. However, there is a growing concern regarding the significant loss of efficiency of these antibiotics against the MRSA strain [7]. The global dissemination of the multidrug-resistant MRSA strain over the past few decades is primarily attributed to the extensive use of vancomycin as a treatment for MRSA infections, which has considerably intensified selective pressure and led to decreased susceptibility to this antibiotic [19]. Hence, there are fears that MRSA may become completely resistant to vancomycin or any other chemotherapy in the future [3,4,20]. Some researchers have investigated combinations of antibiotics that could be effective against MRSA but, unfortunately, most of the resulting data from these combinations are not sufficient to recommend these therapies for critical MRSA infections [7]. Therefore, there is an urgent need to find antibiotics that are effective in treating critical MRSA infections [3,4,20].

MRSA strains are known to contain the major antibiotic resistance gene *mecA* [21]. This gene is responsible for synthesizing a cell wall-forming transpeptidase, penicillin-binding protein (PBP), which is carried on the staphylococcal cassette chromosome *mec* and induces resistance to methicillin and other β-lactam antibiotics [8,22]. The *mecA* gene is not naturally present in *S. aureus* but has been acquired from an external source via an unknown mechanism. In addition to *S. aureus*, *mecA* has been identified in other staphylococcal species originating from humans and animals, including *S. sciuri*, *S. pseudintermedius*, *S. intermedius*, *S. vitulinus*, *S. epidermidis*, *S. haemolyticus*, and *S. saprophyticus* [23]. Some of these staphylococcal species were previously known to cause infections in animals but are now being recognized as a potential threat to human health. Currently, there are two types of *mecA*, *mecA1* and *mecA2*, that have been identified in different staphylococcal species. Other *mec* homologs include *mecB*, *mecC*, and *mecD* [19].

Due to the presence of the *mecA* gene, the options for treating MRSA infections are limited. To combat antibiotic-resistant bacterial infections, new antibiotics have been developed. Unfortunately, resistance to these new antibiotics has already been reported [20]. Therefore, there is an urgent need to search for alternative chemotherapy options for the treatment of MRSA infections, especially in critical conditions.

The process of discovering and developing novel antibiotics for human use is challenging and can take years of laboratory experimentation and thorough clinical trials [14,24]. For several decades, the development of new antibiotics has significantly decreased, partly due to challenges in discovering novel chemotherapy with innovative mechanism(s) of action [24]. Regulatory guidance and decision making also pose significant changes and challenges. To address this issue, various recent initiatives and strategies have been introduced to encourage the development of effective antibiotics for treating antibiotic-resistant infections [25]. Therefore, there is a need to explore already-approved drugs/antibiotics that could be effective against the MRSA strain [9,26]. Repurposing existing drugs can significantly reduce the time and costs associated with conventional drug discovery processes [10,27,28]. The success of this repurposing strategy has been reported in the literature for the identification of new antibacterial drugs [11,12,13]. For instance, 5-fluorouracil and gallium nitrate, which are used in the treatment of cancer and hypercalcemia, respectively, have been found to be effective against certain Gram-negative and Gram-positive pathogens [29,30].

This study focuses on the screening of commercially available approved drugs/antibiotics against the antibiotic resistance protein MecA of methicillin-resistant *S. aureus* (MRSA) strains for potential chemotherapy. This work builds upon our previous study, which identified different antibiotic-resistant genes present in various strains of *S. aureus* [21]. Previously, we conducted a comprehensive analysis of 11 *S. aureus* genome sequences to identify Antibiotic Resistance Genes (ARGs) and discovered 32 such genes, including the mecA gene and other related and vital ARGs. In the current project, computational approaches were utilized to screen drug databases for potential compounds that could inhibit the MecA antibiotic resistance protein of MRSA strains. Two approaches were used to comprehensively search for approved drugs that can inhibit MecA: a protein sequence search and a structural similarity approach, followed by molecular docking experiments. The results obtained from the MecA sequence search suggest that cefoperazone, mezlocillin, cefpiramide, ceftolozane, carindacillin, piperacillin, and ertapenem are strong binders to the MRSA-MecA protein. The structural similarity approach revealed that afamelanotide, an approved drug with unclear antibiotic activity, has a strong binding affinity to the MRSA-MecA protein. This study demonstrates that already-approved drugs have the potential for chemotherapy against antibiotic-resistant pathogens, such as MRSA.

## 2. Materials and Methods

### 2.1. Mining and Downloading of MecA and Blasting against the DrugBank Database

The MecA amino acid sequence in the FASTA Format with an accession number of AGC51118 was obtained from the National Center for Biotechnology Information (NCBI). The sequence was then queried against all approved drugs in the DrugBank database (www.drugbank.com, accessed on 21 February 2023) using the target sequence tool. The search parameters were set to select approved drugs under drug types and target, enzymes, carrier, and transporter as protein types, while leaving other parameters at default settings. As a result, 50 approved compounds/drugs were identified as potential targets for the treatment of bacterial infections, based on significant E-values, bit-score, query length, and alignment length. These compounds were downloaded in pdb format for further analysis.

### 2.2. Modeling and Docking Experiment

The structure of *S. aureus* MecA in the Protein Data Bank exists as a complex with ClpC (PDB number 6EMW) [31], penicillin-binding protein 2a (PDB number 1VQQ) [32], and MecI repressor-operator (PDB number 2D45) [33]. Hence, the structure of MRSA MecA was modeled using the amino acid sequence and the Swiss-Model online tool (https://swissmodel.expasy.org, accessed on 23 February 2023). This tool is a fully automated protein structure homology-modeling server that works via the Expasy web server or through the program DeepView (Swiss Pdb-Viewer) [34,35,36]. The MecA amino acid sequences in the FASTA format were used to build the 3D structure from the structure of nitrocefin acyl-Penicillin binding protein 2a from methicillin-resistant *S. aureus* strain 27r at a 2.00 A resolution [32]. The modeled 3D structure of MecA was obtained with the confidence gradient and DSSP secondary structure selected. Structure validation and quality were accessed using MolProbity (http://molprobity.biochem.duke.edu, accessed on 25 February 2023), a structure validation tool that employs all-atom contact analysis tools and updated geometrical criteria for phi/psi, sidechain rotamer, and Cbeta deviation [37]. To dock the 50 downloaded compounds against the modeled MecA structure, we used the CB-Dock2 online tool (https://cadd.labshare.cn/cb-dock2/php/blinddock.php, accessed on 27 February 2023). This tool integrates cavity detection, docking, and homologous template fitting for more accurate blind docking of protein-ligand complexes [38]. Only compounds that showed a strong affinity with vine scores of −8.5 and below were selected for further analysis.

### 2.3. Search for Structural Similarities to Known Antibiotics and High-Affinity Docking

To identify structural similarities to the gold-standard MRSA antibiotic vancomycin and others, including daptomycin, linezolid, tigecycline, quinupristin/dalfopristin, tetracyclines, rifampin, and gentamicin [7,18], we performed a docking experiment with these known and approved antibiotics against the MecA-modelled structure, as described above. The antibiotics with the highest affinity were selected and a search for structural similarities was completed using the structure similarity tools on the DrugBank database. Drugs that have been approved for the treatment of different diseases were obtained in PDB format for further docking experimentation [38]. The compounds that bind to a MecA-modeled structure with a high affinity were selected for discussion.

## 3. Results

**Figure 1:**
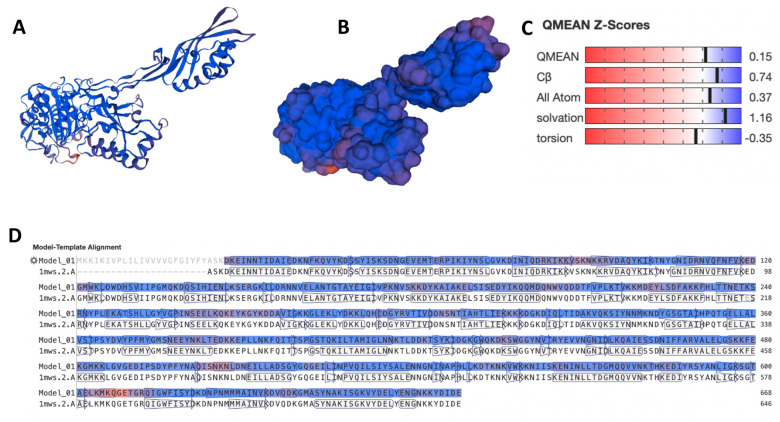
Modeled MRSA−MecA protein structure. (**A**) The carton structure of the modeled MRSA−MecA protein. (**B**) The surface structure of the modeled MRSA−MecA protein. (**C**) QMEAN of the modeled MRSA−MecA protein. (**D**) Model−template alignment of the MecA to the structure of nitrocefin acyl−Penicillin binding protein 2a (1MWS) from the methicillin-resistant *Staphylococcus aureus* strain.

The MRSA-MecA structure was successfully modeled (Figure 1A,B) with high GMQE, QMEANDisCo Global, and QMEAN Z-Scores (Figure 1C). The template used for modeling was penicillin-binding protein 2a Structure of nitrocefin acyl-Penicillin binding protein 2 (1MWS) from methicillin-resistant *S. aureus* strain with a Seq Identity of 99.84% (Figure 1D). Using the DrugBank database, we found 50 approved drugs that bind to 18 different proteins, including MecA, with significant E-values and bit scores (Table 1). Peptidoglycan synthase FtsI and penicillin-binding protein 2B were the targets with the highest ligands, with 18 and 17 interacting drugs, respectively. Some drugs bind to more than one target. Most of the interacting drugs were antibiotics used to treat bacterial infections such as urinary tract infections, blood infections, bone and joint infections, and respiratory infections, among others.

**Figure 2:**
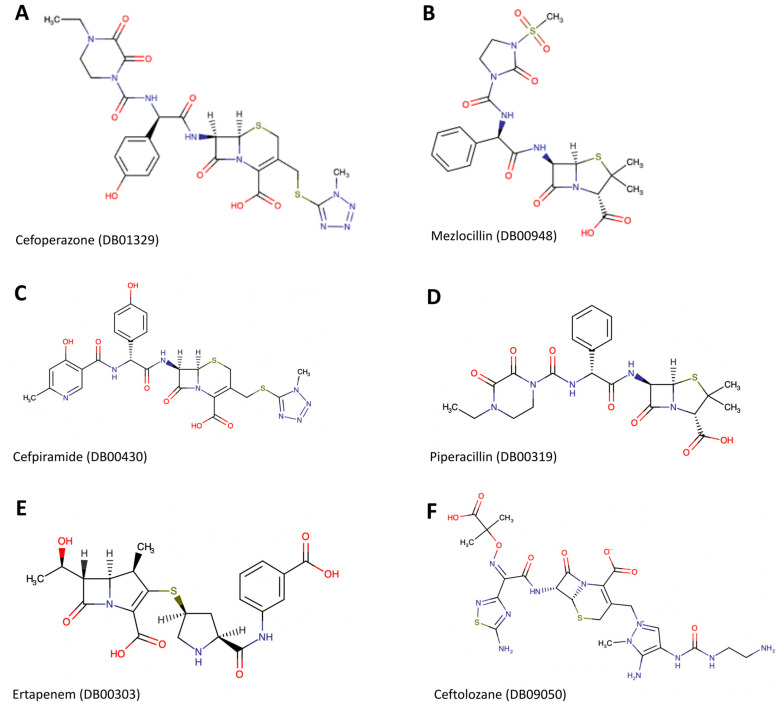
Chemical structure of promising drugs that were obtained from the MecA protein sequence target search.

**Figure 3:**
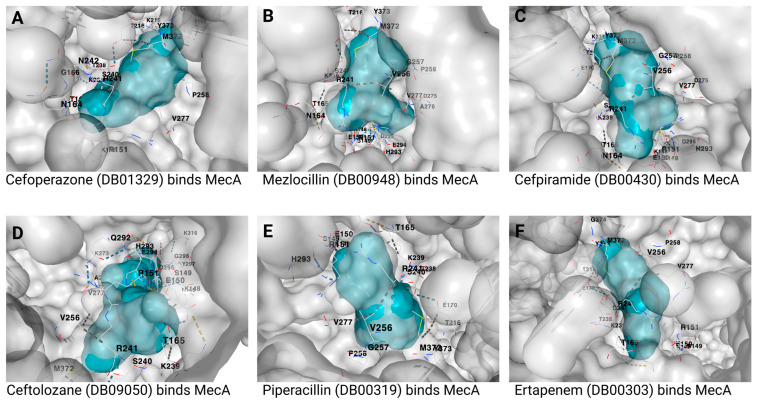
Predicted binding of MecA protein to (**A**) cefoperazone (DB01329), (**B**) mezlocillin (DB00948), (**C**) cefpiramide (DB00430), (**D**) ceftolozane (DB09050), (**E**) ertapenem (DB00303), and (**F**) piperacillin (DB00319). The binding sites of the MecA protein are shown in gray color, whereas the ligands are presented in blue. The DrugBank accession numbers are shown in brackets, whereas the different binding residues of the MecA are written in black.

**Figure 4:**
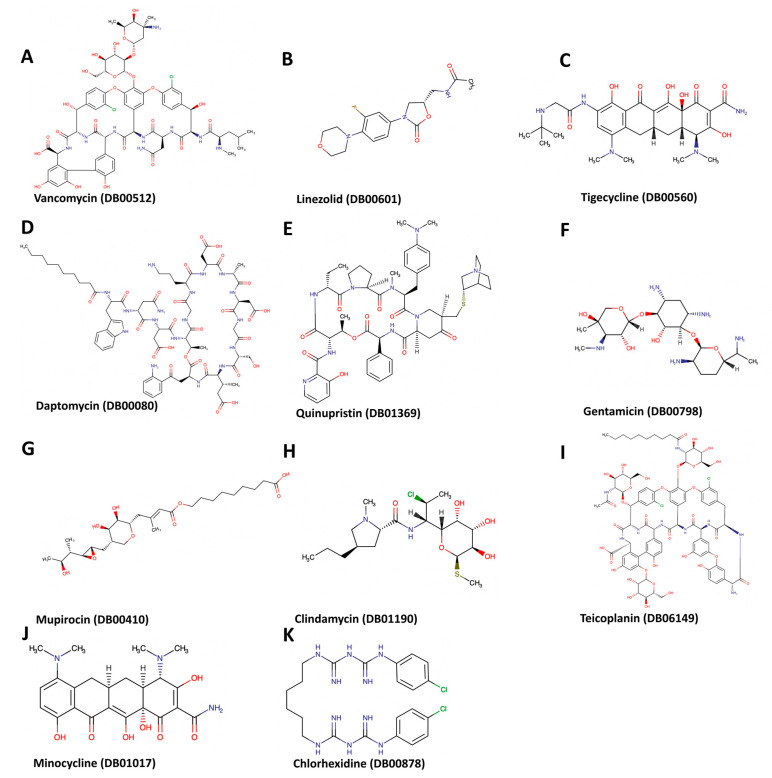
Chemical structures of vancomycin and other *S. aureus* antibiotics as well as drugs that have structural similarities to these known antibiotics.

**Figure 5:**
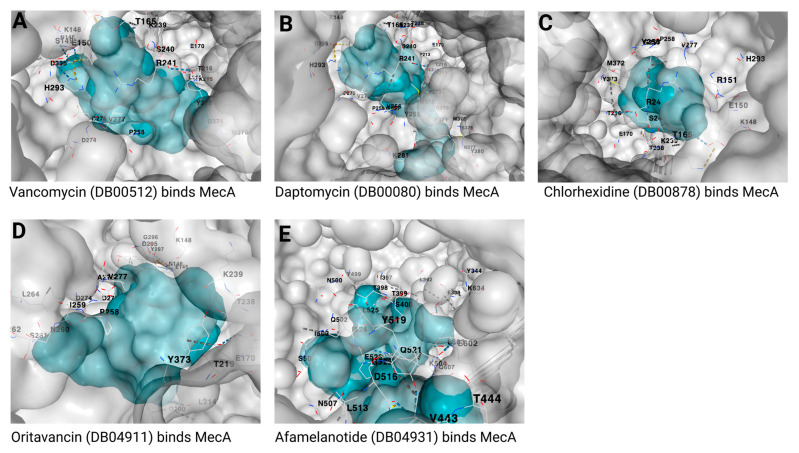
Predicted binding of MecA protein to (**A**) vancomycin, (**B**) daptomycin, (**C**) chlorhexidine, (**D**) oritavancin, and (**E**) afamelanotide. The binding site of the MecA protein is shown in a gray color, whereas the ligands are presented in a blue color. The DrugBank accession numbers are shown in brackets, whereas the different binding residues of the MecA are written in black.

After docking the approved 50 drugs against the MecA protein, only the 6 whose chemical structure is presented in Figure 2A–F have a strong affinity with a vina score of −8.5 or below. These drugs include cefoperazone, mezlocillin, cefpiramide, ceftolozane, piperacillin, and ertapenem with vina scores of −9.3, −8.8, −8.8, −8.7, −8.6, and −8.5, respectively (Table 2). Figure 3 shows the binding structure of these drugs to MecA. The results from the docking experiment of the known antibiotics used in the treatment of MRSA infection show that vancomycin, daptomycin, chlorhexidine, and quinupristin/dalfopristin (Figure 4) have vina scores equal to or lower than −8.5, with vancomycin having the lowest score of −11.6, as presented Figure 5A–C. The results from the structural similarity experiment of these already known and approved antibiotics show that oritavancin and telavancin were the only approved drugs similar in structure to vancomycin, with a score of 0.963 and 0.96, respectively, indicating that these drugs are above 96% similar in structure to vancomycin.

**Figure 6:**
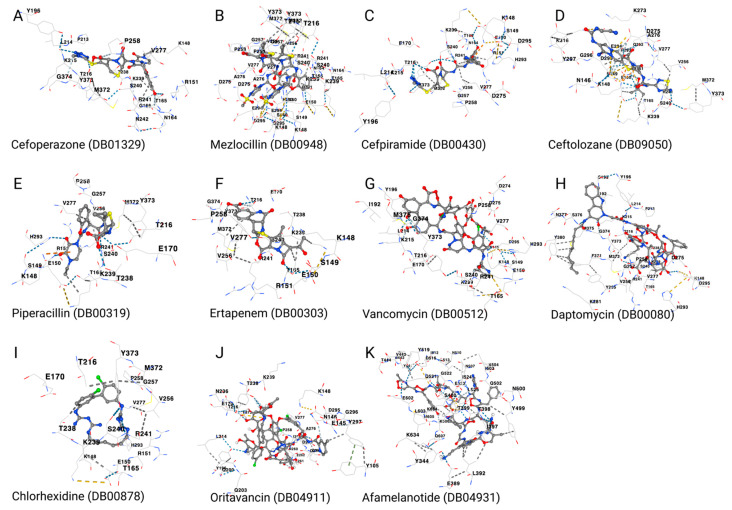
MRSA-MecA binding residues to the different drugs/antibiotics. (**A**) Cefoperazone binds to MecA with the following amino acids: LYS148, ARG151, ASN164, THR165, GLY166, TYR196, PRO213, LEU214, LYS215, THR216, THR238, LYS239, SER240, ARG241, ASN242, PRO258, VAL277, MET372, TYR373, and GLY374. (**B**) Mezlocillin binds to MecA with the following amino acids: LYS148, SER149, GLU150, ARG151, ASN164, THR165, THR216, LYS239, SER240, ARG241, VAL256, GLY257, PRO258, ASP275, ALA276, VAL277, HIS293, GLU294, ASP295, MET372, and TYR373. (**C**) Cefpiramide binds to MecA with the following amino acids: LYS148, SER149, GLU150, ARG151, ASN164, THR165, GLU170, TYR196, LEU214, LYS215, THR216, LYS239, SER240, ARG241, VAL256, GLY257, PRO258, ASP275, VAL277, HIS293, ASP295, MET372, and TYR373. (**D**) Ceftolozane binds to MecA with the following amino acids: ASN146, LYS148, SER149, GLU150, ARG151, THR165, LYS239, SER240, ARG241, VAL256, LYS273, ASP275, ALA276, VAL277, GLN292, HIS293, GLU294, ASP295, GLY296, TYR297, LYS316, MET372, and TYR373. (**E**) Piperacillin binds to MecA with the following amino acids: LYS148, SER149, GLU150, ARG151, THR165, GLU170, THR216, THR238, LYS239, SER240, ARG241, VAL256, GLY257, PRO258, VAL277, HIS293, MET372, and TYR373. (**F**) Ertapenem binds to MecA with the following amino acids: LYS148, SER149, GLU150, ARG151, THR165, GLU170, THR216, THR238, LYS239, SER240, ARG241, VAL256, PRO258, VAL277, MET372, TYR373, and GLY374. (**G**) Vancomycin binds to MecA with the following amino acids: ASN146, LYS148, SER149, GLU150, THR165, GLU170, ILE192, TYR196, LEU214, LYS215, THR216, LYS239, SER240, ARG241, PRO258, ASP274, ASP275, VAL277, HIS293, ASP295, TYR373, GLY374, and MET375. (**H**) Daptomycin binds to MecA with the following amino acids: Chain A: LYS148, THR165, GLU170, ILE192, SER193, TYR196, PRO213, LEU214, LYS215, THR216, THR238, LYS239, SER240, ARG241, TYR255, VAL256, GLY257, PRO258, ASP275, VAL277, LYS281, HIS293, ASP295, PHE371, MET372, TYR373, GLY374, MET375, SER376, ASN377, and TYR380. (**I**) Chlorhexidine binds to MecA with the following amino acids: LYS148, GLU150, ARG151, THR165, GLU170, THR216, THR238, LYS239, SER240, ARG241, VAL256, GLY257, PRO258, VAL277, HIS293, MET372, and TYR373. (**J**) Oritavancin binds to MecA with the following amino acids: TYR105, GLU145, ASN146, LYS148, GLU170, TYR196, GLN200, GLN203, LEU214, THR216, ASN236, THR238, LYS239, PRO258, ILE259, ASN260, SER261, GLU262, LEU264, ASP274, ASP275, ALA276, VAL277, ASP295, GLY296, TYR297, and TYR373. (**K**) Afamelanotide binds to MecA with the following amino acids: TYR344, GLU389, LEU392, ILE397, THR398, THR399, SER400, TYR441, ASN442, VAL443, THR444, TYR499, ASN500, GLN502, ILE503, SER504, ASN507, ASN510, ILE512, LEU513, ASP516, TYR519, GLN521, GLY522, GLU523, ILE524, LEU525, GLU602, LEU603, LYS604, MET605, LYS606, GLN607, and LYS634. The drug structure is represented by ball and stick. The DrugBank accession numbers are shown in brackets, whereas the different binding residues of the MecA are written in black.

The structure with the greatest similarity to daptomycin was afamelanotide, with a similarity score of 0.703. As for chlorhexidine, the only approved drug found to be structurally similar was proguanil, with a score of 0.784. We found no approved drug with structural similarity to quinupristin/dalfopristin. When we docked these drugs to the MecA protein, we found that oritavancin, telavancin, afamelanotide, and proguanil had a vina score of −10.3, −8.4, −9.5, and −6.7, respectively (Figure 5D,E and Table 3). Hence, all structurally similar drugs had a significant binding affinity, except for proguanil. Figure 6 shows the interactions between the amino acid residues of the MecA protein and each drug/antibiotic used in this study.

## 4. Discussion

The MRSA strain is responsible for causing a large number of infections acquired in hospitals and communities, which can have severe consequences [1,9,16,39,40]. This strain has several virulence factors and toxins that often contribute to toxin-mediated diseases, including toxic shock syndrome, staphylococcal foodborne diseases, and scalded skin syndrome [22,40]. One significant challenge associated with MRSA is its high resistance to multiple antibiotic classes, making treatment complicated [1,9,40]. MRSA strains have an altered penicillin-binding protein (PBP) that reduces their affinity for most semisynthetic penicillins. The PBP protein is encoded by an acquired gene, mecA, which is found in various bacterial species. Different mecA homologs have been found in different species and are grouped into prototype types based on their nucleotide sequence similarity, including *mecA*, *mecB*, and *mecC*, reflecting their order of discovery [40]. However, the *mec* nomenclature system is not exclusive to the *Staphylococcus* genus, as these genes are present on mobile genetic elements and can be found outside of specific species or genera [40,41]. In addition, MRSA colonization and infection have been increasingly prevalent in various ecological niches. Since the 1990s, countries worldwide have reported a rise in community-associated MRSA infections. Livestock-associated MRSA, which infects both livestock and the humans in contact with these animals, has been identified in several countries in recent years. Reports have also shown the interplay between these distinct MRSA reservoirs, such as nosocomial infections caused by community-associated MRSA and the transfer of livestock-associated MRSA into hospitals [39].

Therefore, it is crucial to look for alternatives to currently available antibiotics for the treatment of MRSA infections [40,42,43]. When compared to conventional drug research and development methods, the repurposing of already-approved medication tactics against known pathogen targets is strongly advocated [10,11,12,27,28,44,45,46]. The goal of this initiative is to look for *mecA*-resistant *S. aureus* (MRSA) strains that are resistant to already-approved medicines and treatments. Methicillin, penicillin, and other penicillin-like drug resistance is caused by the MRSA bacteria strains expressing the *mecA* antibiotic resistance gene [41]. Following MecA protein sequence interrogation, drugs were retrieved from the drug database that are known to inhibit the action of various isoforms of penicillin-binding protein, which is one of the key factors in MRSA resistance to antibiotics [47]. Research has shown that the PBPs are protein-mediated through the *mecA* gene to enhance MRSA resistance to all the β-lactam antibiotics [22,41]. The majority of the medications obtained using this method are antibiotics that fight various pathogenic bacteria known to cause life-threatening diseases.

Six authorized medications were found to have a high binding affinity against the MecA protein through the sequence interaction and docking experiment on the MecA protein. These medicines are well-known antibiotics that are used to treat various bacterial infections. Cefoperazone, a cephalosporin antibiotic used to treat bacterial infections, was the drug with the highest binding affinity. Cefoperazone was initially thought to be useful in treating *Pseudomonas* infections, but it is also useful in treating a number of diseases brought on by susceptible pathogenic bacteria in the body [48,49]. It is well-known in the scientific community that cefoperazone binds to certain penicillin-binding proteins (PBPs) in the bacterial cell wall, inhibits cell wall production, and promotes the activity of lysis autolytic enzymes, such as autolysins [48]. Cefoperazone has also been demonstrated to prevent *Escherichia coli* (strain K12) peptidoglycan synthase FtsI and D-alanyl-D-alanine carboxypeptidase Dac proteins from being activated [49]. The outcomes of this study further demonstrate that cefoperazone could bind to the MRSA-MecA protein; however, in vivo biochemical works need to be carried out to confirm this affinity. Mezlocillin and cefpiramide, which are semi-synthetic antibiotics effective against some pathogenic bacteria and share the same mode of action as cefoperazone, were also discovered to have intriguing compounds that strongly bind to the MecA protein [50,51]. These broad-spectrum third-generation antibiotics are known to be effective against *Pseudomonas aeruginosa* and other pathogenic bacteria that are comparable [51]. We also discovered that ceftolozane, which is well-known to be highly efficient against complex infections [52,53], was another potent ligand of MecA proteins. Mezlocillin is known to be stable against hydrolysis by a variety of beta-lactamases, including penicillinases and cephalosporinases, and is used to treat infections caused by susceptible strains of *H. influenzae*, *Klebsiella species*, *Pseudomonas species*, *Proteus mirabilis*, *E. coli*, *Enterobacter species*, *Streptococcus faecelis*, *Peptococcus species*, and *Peptostreptococcus species* [50].

It was shown that piperacillin, a semi-synthetic, broad-spectrum antibiotic that is related to penicillins and beta-lactamase inhibitors [54], also has a considerable affinity for MecA proteins. Because it binds to particular PBPs in bacterial cell walls, piperacillin is used as a beta-lactamase inhibitor in combination with tazobactam to treat severe infections brought on by MRSA [55]. Piperacillin is effective in the treatment of bacterial infections caused by susceptible Gram-positive and Gram-negative organisms. Piperacillin is stable against hydrolysis by a variety of beta-lactamases and is eliminated primarily by glomerular filtration and tubular secretion. It is also excreted by the biliary route and can be safely used in patients with severely restricted kidney function [54,55]. Ertapenem, a carbapenem antibiotic used to treat moderate to severe infections, was the final product of this method [56,57]. The ertapenem antibiotic, like the other five antibiotics mentioned, binds to bacterial PBPs to prevent and facilitate the lysing of the cell wall [56]. Ertapenem is used for the treatment of moderate to severe infections caused by susceptible bacteria. It is effective against a wide range of Gram-positive and Gram-negative aerobic and anaerobic bacteria. Ertapenem is indicated for the treatment of complicated intra-abdominal infections, complicated skin and skin structure infections, community-acquired pneumonia, complicated urinary tract infections, acute pelvic infections, acute gynecological infections, and prophylaxis of surgical site infection following elective colorectal surgery. The possibility that these six antibiotics could disrupt the activity of the MecA protein as a mode of action to prevent bacterial growth is one of this study’s most important findings.

Docking experiments and structural similarity to known *S. aureus* antibiotics were conducted to identify which of the drugs would bind robustly to the MecA protein. Vancomycin strongly binds to MecA proteins, as expected. Vancomycin is the current antibiotic of choice for treating MRSA infections. It is derived from *Streptomyces orientalis* and works by inhibiting the assembly of bacterial cell walls, similar to the approved medications discussed earlier. Intravenous administration is used to treat septicemia, infective endocarditis, and other infections, whereas oral administration is employed to treat *Clostridium difficile*-associated diarrhea and enterocolitis caused by *Staphylococcus aureus* [3,20]. Daptomycin and chlorhexidine are two other substances that bind firmly. Daptomycin is a cyclic lipopeptide antibiotic used to treat bacterial infections, including skin and soft tissue infections, bacteremia, and endocarditis. It is effective against a variety of Gram-positive bacteria, including MRSA and VRE. Daptomycin works by disrupting bacterial membrane function, leading to bacterial cell death [58]. Chlorhexidine, on the other hand, is an antiseptic and disinfectant that is commonly used to prevent and treat infections. It is effective against a wide range of microorganisms, including Gram-positive and Gram-negative bacteria, fungi, and viruses. Chlorhexidine is often used as a skin disinfectant before surgery or other medical procedures and can also be used as a mouthwash to prevent or treat oral infections [59].

Oritavancin shared a considerable structural similarity with vancomycin and exhibited a high affinity for the MecA protein. Oritavancin is also used to treat infections caused by enterococci, streptococci, MRSA, and *S. aureus* [60]. Pentaglycyl bridging segments, bacterial cell wall peptide bridging segments, and peptidoglycan precursors are all inhibited by oritavancin [61,62]. Once more, we demonstrate in the present study that oritavancin might bind to MecA proteins. Afamelanotide, the only medication licensed for the treatment of erythropoietic protoporphyria and a first-in-class synthetic 13-amino acid peptide analog of the endogenous alpha-melanocyte-stimulating hormone, shared structural similarities with daptomycin [63,64]. By attaching to the melanocortin-1 receptor (MC1R) on melanocytes, afamelanotide facilitates the manufacture of eumelanin, a photoprotective substance [64,65]. When afamelanotide promotes MC1R signaling, there are other known protective activities such as enhanced antioxidant activity, DNA repair, and production of immunomodulatory proteins including interleukin-10 [64]. Furthermore, there is evidence that treatment with afamelanotide, which was approved by the European Union in early 2015 and by the United States in early 2019, significantly enhances the quality of life for patients [66].

## 5. Conclusions

In order to find brand-new, FDA-approved medications for the treatment of MRSA infection, we used a sequence binding approach and a structural similarity strategy. Our work provides evidence supporting the potential for several antibiotics to be utilized as alternatives to the current class of antibiotics used for treating MRSA. Six approved medications, including cefoperazone, mezlocillin, cefpiramide, ceftolozane, piperacillin, and ertapenem, exhibited a high binding affinity against the MecA protein. A key discovery from this research is that the antibiotic compound afamelanotide shows promise for treating MRSA infection and other related bacterial diseases because of its high affinity for MecA proteins. Therefore, a lab experiment is necessary to confirm the inhibitory effects of afamelanotide and the other drugs identified in this project that binds to the MRSA-MecA protein in silico.

## Figures and Tables

**Table 1:** MecA sequence targets after searching the DrugBank dataset.

Target	Protein	E Value	Bit Score	Query Length	Alignment Length	No. of Binding Drugs to Each Target
1	MecA	0	1273.46	668	645	1
2	MecA PBP2′ (penicillin binding protein 2′)	0	1216.83	668	649	2
3	Penicillin binding protein 2a	0	979.934	668	486	4
4	Penicillin-binding protein 3	1.39 × 10^−132^	404.06	668	532	4
5	Penicillin-binding protein 3	2.63 × 10^−121^	375.17	668	609	1
6	Penicillin-binding protein 2	6.14 × 10^−62^	199.904	668	95	1
7	Penicillin-binding protein 2	9.59 × 10^−43^	162.54	668	553	2
8	Penicillin-binding protein 2	1.19 × 10^−38^	150.599	668	515	17
9	Penicillin-binding protein 2	7.65 × 10^−35^	139.043	668	564	2
10	Penicillin-binding protein 2	7.69 × 10^−31^	126.716	668	530	2
11	Peptidoglycan synthase FtsI	1.88 × 10^−20^	94.3597	668	556	18
12	Peptidoglycan synthase FtsI	2.91 × 10^−19^	90.5077	668	551	1
13	PBP3	1.72 × 10^−17^	85.1149	668	551	1
14	Penicillin-binding protein 2	3.71 × 10^−16^	80.4925	668	457	1
15	Cell division protein	1.28 × 10^−15^	78.9518	668	515	4
16	Penicillin-binding protein 2x	3.20 × 10^−15^	77.7962	668	522	2
17	Penicillin-binding protein 2X	5.65 × 10^−15^	77.0258	668	522	2
18	Penicillin-binding protein 2B	6.89 × 10^−12^	67.0106	668	462	2
19	Penicillin-binding protein 2B	6.89 × 10^−12^	67.0106	668	462	17

**Table 2:** Drugs with the highest binding affinity to MRSA-MecA proteins.

S/N	Generic Name	DrugBank Accession Number	Vina Score	Cavity Volume (Å3)	Center (x, y, z)	Docking Size (x, y, z)
1	Cefoperazone	DB01329	−9.3	886	−3, 43, 37	27, 27, 27
2	Mezlocillin	DB00948	−8.8	886	−3, 43, 37	23, 23, 23
3	Cefpiramide	DB00430	−8.8	886	−3, 43, 37	26, 26, 26
4	Ceftolozane	DB09050	−8.7	886	−3, 43, 37	27, 27, 27
5	Piperacillin	DB00319	−8.6	886	−3, 43, 37	24, 24, 24
6	Ertapenem	DB00303	−8.5	886	−3, 43, 37	23, 23, 23

**Table 3:** MRSA-MecA binding affinity to some known *S. aureus* antibiotics as well as the structural similarity of the drugs.

S/N	Generic Name	DrugBank Accession Number	Vina Score	Cavity Volume (Å3)	Center (x, y, z)	Docking Size (x, y, z)
1	Daptomycin	DB00080	−9.8	886	−3, 43, 37	34, 34, 34
2	Linezolid	DB00601	−7.2	886	−3, 43, 37	24, 24, 24
3	Tigecycline	DB00560	−8.4	886	−3, 43, 37	26, 26, 26
4	Quinupristin/Dalfopristin	DB01369	−8.5	863	−19, 38, 32	28, 28, 28
5	Vancomycin	DB00512	−11.6	886	−3, 43, 37	32, 32, 32
6	Gentamicin	DB00798	−7.6	886	−3, 43, 37	24, 24, 24
7	Mupirocin	DB00410	−7.5	886	−3, 43, 37	32, 32, 32
8	Minocycline	DB01017	−7	996	−33, 53, 59	22, 22, 22
9	Clindamycin	DB01190	−6.5	886	−3, 43, 37	22, 22, 22
10	Chlorhexidine	DB00878	−9.3	886	−3, 43, 37	22, 22, 22
11	Oritavancin	DB04911	−10.3	886	−3, 43, 37	34, 34, 34
12	Telavancin	DB06402	−8.4	863	−19, 38, 32	34, 34, 34
13	Afamelanotide	DB04931	−9.5	886	−3, 43, 37	38, 38, 38
14	Proguanil	DB01131	−6.7	996	−33, 53, 59	22, 22, 22

## Data Availability

All data underlying the results are included as part of the published article.
